# Supplementary material for: Compositional Lotka-Volterra describes microbial dynamics in the simplex
Source: PLoS Comput Biol. 2020 May 29;16(5):e1007917. doi: 10.1371/journal.pcbi.1007917 (PMC7325845; doi:10.1371/journal.pcbi.1007917)
Supplement: S1 Appendix — (PDF) [file pcbi.1007917.s001.pdf]

Appendix to Compositional Lotka-Volterra describes microbial dynamics in the simplex

Tyler A. Joseph[1], Liat Shenhav[2], Joao B. Xavier[3], Eran Halperin[2,4,5], Itsik Pe'er[1,6,7*]

1 Department of Computer Science, Columbia University, New York, NY, USA
2 Department of Computer Science, UCLA, Los Angeles, CA, USA
3 Memorial Sloan Kettering Cancer Center, New York NY, USA
4 Department of Human Genetics, University of California Los Angeles, Los Angeles, CA, USA
5 Department of Anesthesiology and Perioperative Medicine, University of California Los Angeles, Los Angeles, CA, USA
6 Department of Systems Biology, Columbia University, New York, NY, USA
7 Data Science Institute, Columbia University, New York, NY, USA

* itsik@cs.columbia.edu

Appendix A: Changing the denominator of the additive log-ratio transformation

Given parameters with respect to one choice of denominator of the additive log-ratio, say $\pi_D(t)$, we can obtain parameters for any other choice of denominator. For instance, if we are interested in the relationship between species i and j, we can express how their log ratio changes over time as follows:

$$\frac{d}{dt}\log\left(\frac{\pi_i(t)}{\pi_j(t)}\right) = \frac{d}{dt}\log\left(\frac{\pi_i(t)}{\pi_D(t)}\right) - \frac{d}{dt}\log\left(\frac{\pi_j(t)}{\pi_D(t)}\right) \tag{1}$$

$$= (g_i - g_j) + \sum_{k=1}^{D}(A_{ik} - A_{jk})\pi_k(t) + \sum_{p=1}^{P}(B_{ip} - B_{jp})u_p(t) \tag{2}$$

Appendix B: Solving for $\frac{d}{dt}\pi_i(t)$

We can also express cLV as a dynamical system in terms of derivatives with respect to relative abundances $\frac{d}{dt}\pi_i$ for $i = 1, ...D$. First, note

$$\pi_D = 1 - \sum_{j=1}^{D-1}\pi_j \tag{3}$$

$$\implies \frac{d}{dt}\pi_D = -\sum_{j=1}^{D-1}\frac{d}{dt}\pi_j \tag{4}$$

where the π_i are implicit functions of t. Next, using $\frac{d}{dt}\log\pi_i = \frac{\frac{d}{dt}\pi_i}{\pi_i}$ we have

$$\frac{d}{dt}\log\left(\frac{\pi_i}{\pi_D}\right) = \frac{d}{dt}\log\pi_i - \frac{d}{dt}\log\pi_D \tag{5}$$

$$= \frac{\frac{d}{dt}\pi_i}{\pi_i} - \frac{\frac{d}{dt}\pi_D}{\pi_D} \tag{6}$$

$$= \frac{\frac{d}{dt}\pi_i}{\pi_i} - \frac{\frac{d}{dt}1 - \sum_j \pi_j}{\pi_D} \tag{7}$$

$$= \frac{\frac{d}{dt}\pi_i}{\pi_i} + \sum_j \frac{\frac{d}{dt}\pi_j}{\pi_D} \tag{8}$$

$$= \frac{d}{dt}\pi_i\left(\frac{1}{\pi_i}\right) + \sum_{j=1}^{D-1}\frac{d}{dt}\pi_j\left(\frac{1}{\pi_D}\right) \tag{9}$$

This allows us to write the derivatives $\frac{d}{dt}\pi_i$ as the solution to the following system of equations.

$$\left(\begin{bmatrix} \frac{1}{\pi_1} & 0 & \cdots & 0 \\ 0 & \frac{1}{\pi_2} & \cdots & 0 \\ \vdots & \vdots & \ddots & \vdots \\ 0 & 0 & \cdots & \frac{1}{\pi_{D-1}} \end{bmatrix} + \frac{1}{\pi_D}\begin{bmatrix} 1 & 1 & \cdots & 1 \\ 1 & 1 & \cdots & 1 \\ \vdots & \vdots & \ddots & \vdots \\ 1 & 1 & \cdots & 1 \end{bmatrix}\right)\begin{bmatrix} \frac{d}{dt}\pi_1 \\ \frac{d}{dt}\pi_2 \\ \vdots \\ \frac{d}{dt}\pi_{D-1} \end{bmatrix} = \begin{bmatrix} \frac{d}{dt}\log\left(\frac{\pi_1}{\pi_D}\right) \\ \frac{d}{dt}\log\left(\frac{\pi_2}{\pi_D}\right) \\ \vdots \\ \frac{d}{dt}\log\left(\frac{\pi_{D-1}}{\pi_D}\right) \end{bmatrix} \tag{10}$$

This system has the following solution

$$\begin{bmatrix} \frac{d}{dt}\pi_1 \\ \frac{d}{dt}\pi_2 \\ \vdots \\ \frac{d}{dt}\pi_{D-1} \end{bmatrix} = \left(\text{diag}\left[\frac{1}{\pi_1}, ..., \frac{1}{\pi_{D-1}}\right] + \frac{1}{\pi_D}\mathbf{1}_{D-1}\mathbf{1}_{D-1}^T\right)^{-1} \begin{bmatrix} \frac{d}{dt}\log\left(\frac{\pi_1}{\pi_D}\right) \\ \frac{d}{dt}\log\left(\frac{\pi_2}{\pi_D}\right) \\ \vdots \\ \frac{d}{dt}\log\left(\frac{\pi_{D-1}}{\pi_D}\right) \end{bmatrix} \tag{11}$$

where we have written the two matrices of the previous equation with a more suggestive form. Applying the Sherman-Morrison formula allows us to write the inverse in closed form.

$$\left(\text{diag}\left[\frac{1}{\pi_1}, ..., \frac{1}{\pi_{D-1}}\right] + \frac{1}{\pi_D}\mathbf{1}_{D-1}\mathbf{1}_{D-1}^T\right)^{-1} = \text{diag}\left[\pi_1, ..., \pi_{D-1}\right] - \begin{bmatrix} \pi_1 \\ \pi_2 \\ \vdots \\ \pi_{D-1} \end{bmatrix} \begin{bmatrix} \pi_1 \\ \pi_2 \\ \vdots \\ \pi_{D-1} \end{bmatrix}^T \tag{12}$$

Plugging in the terms for $\frac{d}{dt}\log\left(\frac{\pi_i}{\pi_D}\right)$ leaves us with the following form for $\frac{d}{dt}\pi_i$

$$\frac{d}{dt}\pi_i = \pi_i\left(\overline{g}_i + \sum_{j=1}^{D}\overline{A}_{ij}\pi_j + \sum_{p=1}^{P}\overline{B}_{ip}u_p\right) - \pi_i\left[\sum_{k=1}^{D-1}\pi_k\left(\overline{g}_k + \sum_{j=1}^{D}\overline{A}_{kj}\pi_j + \sum_{p=1}^{P}\overline{B}_{kp}u_p\right)\right] \tag{13}$$

$$\frac{d}{dt}\pi_D = -\pi_D\left[\sum_{k=1}^{D-1}\pi_k\left(\overline{g}_k + \sum_{j=1}^{D}\overline{A}_{kj}\pi_j + \sum_{p=1}^{P}\overline{B}_{kp}u_p\right)\right] \tag{14}$$

Appendix C: Compositional Lotka-Volterra under the isometric log-ratio transformation

We noted in the main text that we could define a compositional version of gLV under other compositional data transformations. Here, we demonstrate a dynamical system based on the isometric log-ratio transformation (ilr). First, we define column vector

$$\text{alr}(\pi(t)) = \begin{bmatrix} \frac{\pi_i(t)}{\pi_D(t)} & \cdots & \frac{\pi_{D-1}(t)}{\pi_D(t)} \end{bmatrix}^T \tag{15}$$

Egozcue et al. (2003) show that the additive log-ratio transformation can be expressed as a linear transformation of the ilr transformation.

$$\text{alr}(\pi(t)) = F^T U^T \,\text{ilr}(\pi(t)) \tag{16}$$

where

$$U^T = \begin{bmatrix} \uparrow & \cdots & \uparrow \\ u_1 & \cdots & u_{D-1} \\ \downarrow & \cdots & \downarrow \end{bmatrix} \in \mathbb{R}^{D\times(D-1)} \text{ with } u_i^T u_j = \delta_{ij} \tag{17}$$

$$F^T = \begin{bmatrix} I_{D-1} & -\mathbf{1}_{D-1} \end{bmatrix} \in \mathbb{R}^{(D-1)\times D}. \tag{18}$$

Here we use F^T and U^T since Egozcue et al. (2003) use row vectors instead of column vectors—the transpose ensures consistency between the different notations. The vectors u_i arise from a choice of basis, $e_i \in \mathcal{S}^D$, in the simplex under the Aitchison geometry that defines the isometric log-ratio transformation. If we substitute the alr for an equivalent representation of the ilr, we get a new system

$$\frac{d}{dt}\text{alr}(\pi(t)) = \overline{g} + \overline{A}\pi(t) + \overline{B}u(t) = F^T U^T \frac{d}{dt}\text{ilr}(\pi(t)) \tag{19}$$

Note that $F^T U^T \in \mathbb{R}^{(D-1)\times(D-1)}$ is invertible. Again following Egozcue et al. (2003), define the Moore-Penrose generalized inverse of F

$$H = \frac{1}{D}\begin{bmatrix} D-1 & -1 & -1 & \cdots & -1 & -1 \\ -1 & D-1 & -1 & \cdots & -1 & -1 \\ \vdots & \vdots & \vdots & \ddots & \vdots & \vdots \\ -1 & -1 & D-1 & \cdots & D-1 & -1 \end{bmatrix} \in \mathbb{R}^{(D-1)\times D} \tag{20}$$

Then $FH = I_{D-1}$, and $(UF)(HU^T) = UI_{D-1}U^T = UU^T = I_{D-1}$, which implies $(UF)^{-1} = HU^T$. Hence $(F^T U^T)^{-1} = (UF)^{-T} = (HU^T)^T = UH^T$. This gives us

$$\frac{d}{dt}\mathrm{ilr}(\pi(t)) = UH^T\overline{g} + UH^T\overline{A}\pi(t) + UH^T\overline{B}u(t) \tag{21}$$

$$:= g_{\mathrm{ilr}} + A_{\mathrm{ilr}}\pi(t) + B_{\mathrm{ilr}}u(t) \tag{22}$$

Given g_{ilr}, A_{ilr}, and B_{ilr}, we can directly solve for the relative parameters $\overline{g}$, $\overline{A}$, and $\overline{B}$ which correspond to the relative parameters of gLV.

$$\overline{g} = F^T U^T g_{\mathrm{ilr}} \tag{23}$$

$$\overline{A} = F^T U^T A_{\mathrm{ilr}} \tag{24}$$

$$\overline{B} = F^T U^T B_{\mathrm{ilr}}. \tag{25}$$

References

J. J. Egozcue, V. Pawlowsky-Glahn, G. Mateu-Figueras, and C. Barcelo-Vidal. Isometric logratio transformations for compositional data analysis. *Mathematical Geology*, 35(3):279–300, 2003.
