# Supplementary figures and images for: Compositional Lotka-Volterra describes microbial dynamics in the simplex
Source: PLoS Comput Biol. 2020 May 29;16(5):e1007917. doi: 10.1371/journal.pcbi.1007917 (PMC7325845; doi:10.1371/journal.pcbi.1007917)

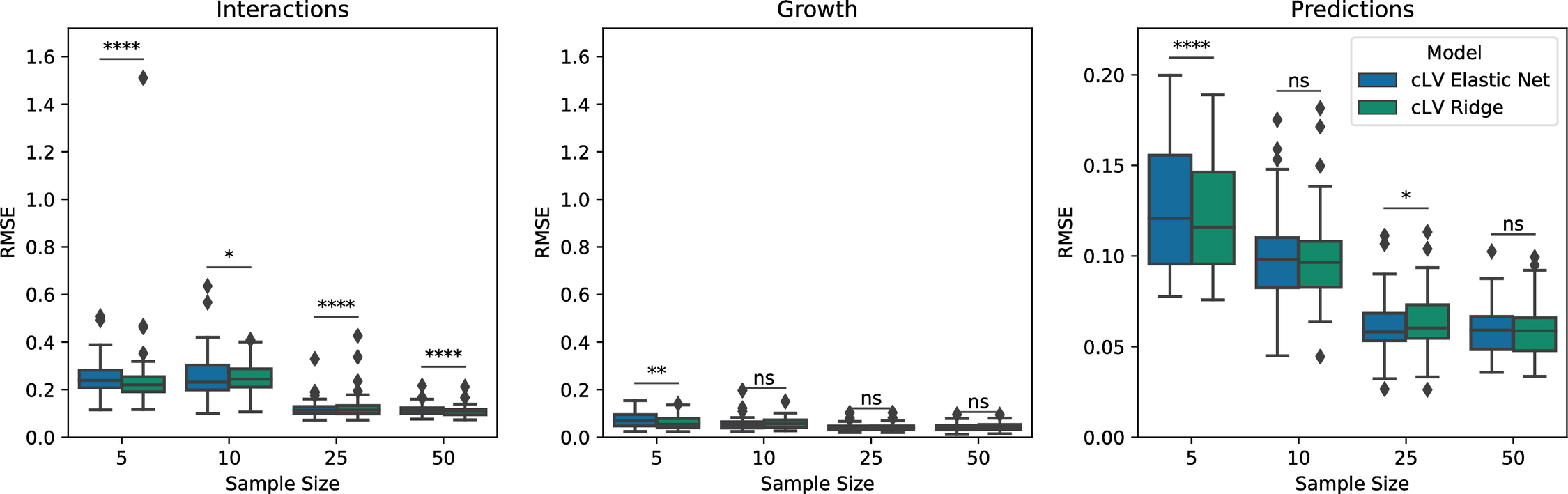

Supplement: S1 Fig — Root-mean-square-error (RMSE; y-axis) between ground truth and estimated interactions, ground truth and estimated growth rates, and predicted trajectories from initial conditions on held out data across 50 simulation replicates. Community trajectories were simulated under cLV, then noisy sequencing counts to with depth of 25000 reads per sample. (TIF) [file pcbi.1007917.s002.tif]

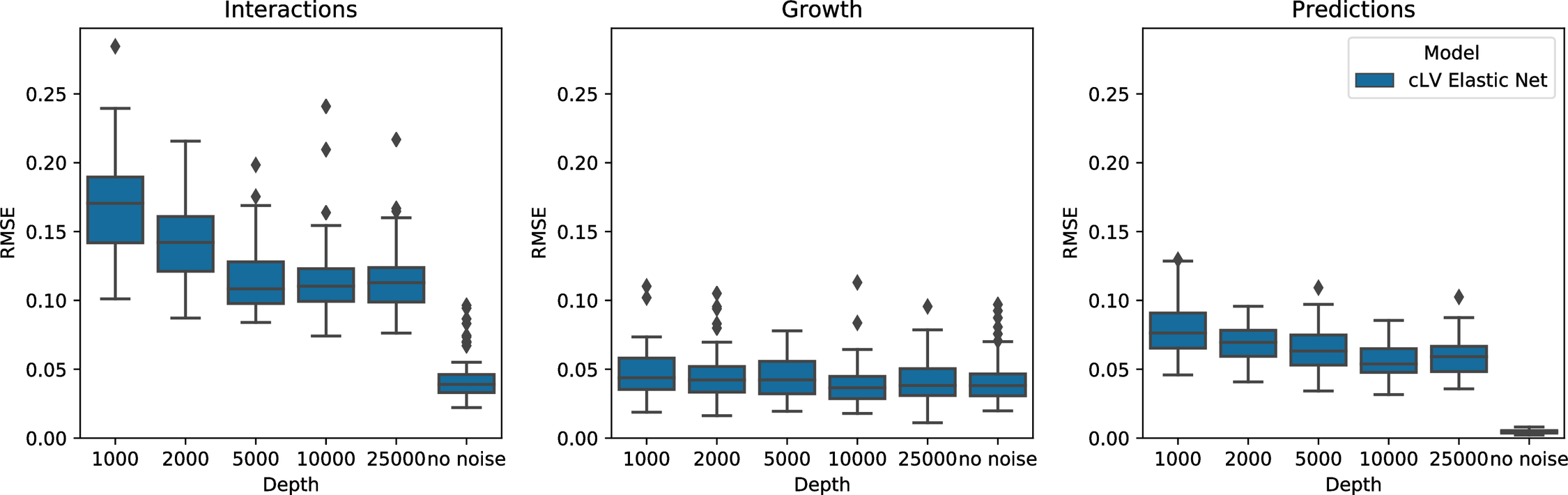

Supplement: S2 Fig — Root-mean-square-error (RMSE; y-axis) between ground truth and estimated interactions, ground truth and estimated growth rates, and predicted trajectories from initial conditions on held out data across 50 simulation replicates. Community trajectories were simulated under cLV, then noisy sequencing counts with increasing sequencing depth. (TIF) [file pcbi.1007917.s003.tif]

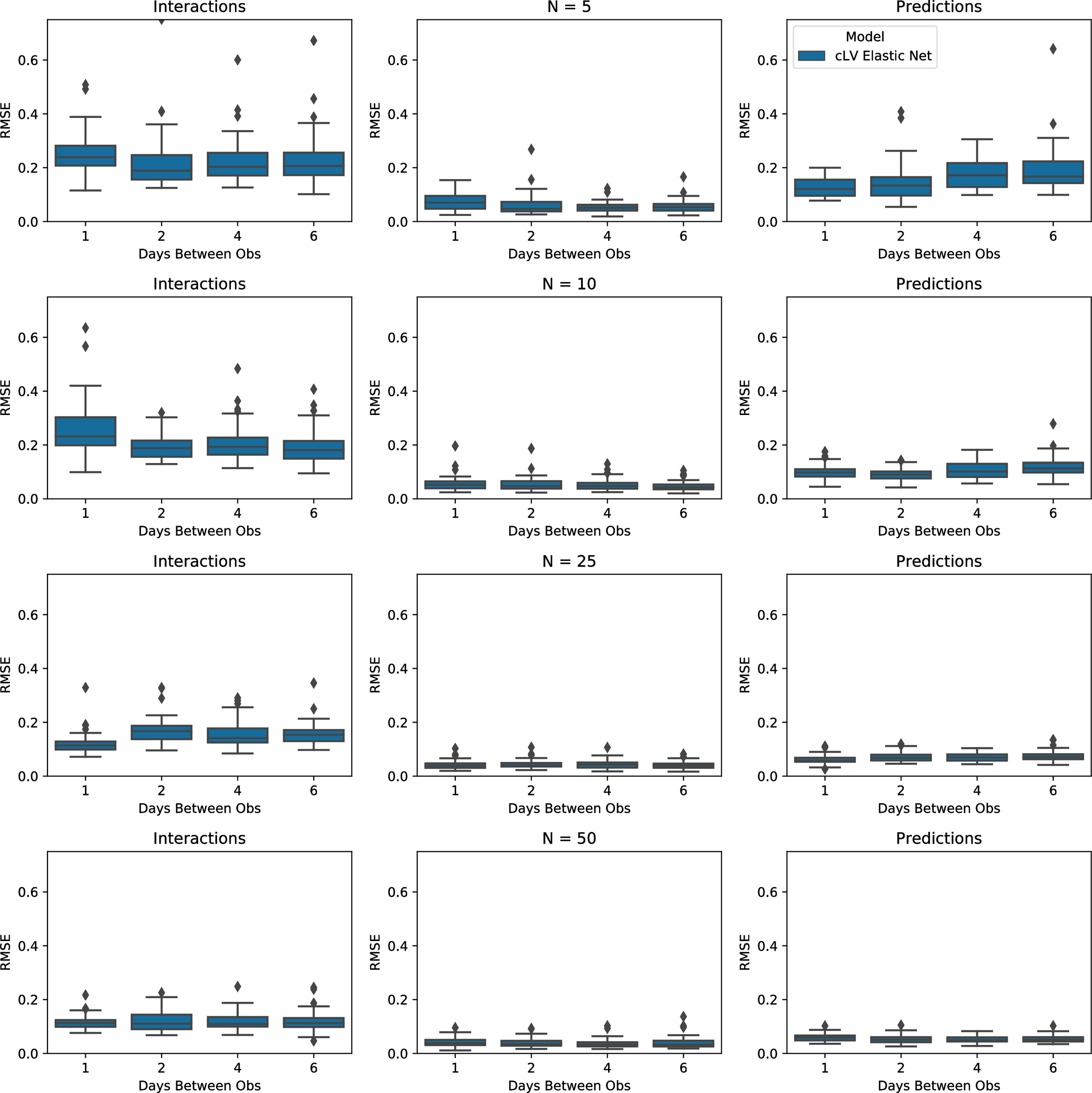

Supplement: S3 Fig — Root-mean-square-error (RMSE; y-axis) between ground truth and estimated interactions, ground truth and estimated growth rates, and predicted trajectories from initial conditions on held out data across 50 simulation replicates. Community trajectories were simulated under cLV. Observations were selected from simulated sequenced space 1, 2, 4, or 6 days apart. Noisy sequencing counts were simulated with a depth of 25000 reads. (TIF) [file pcbi.1007917.s004.tif]

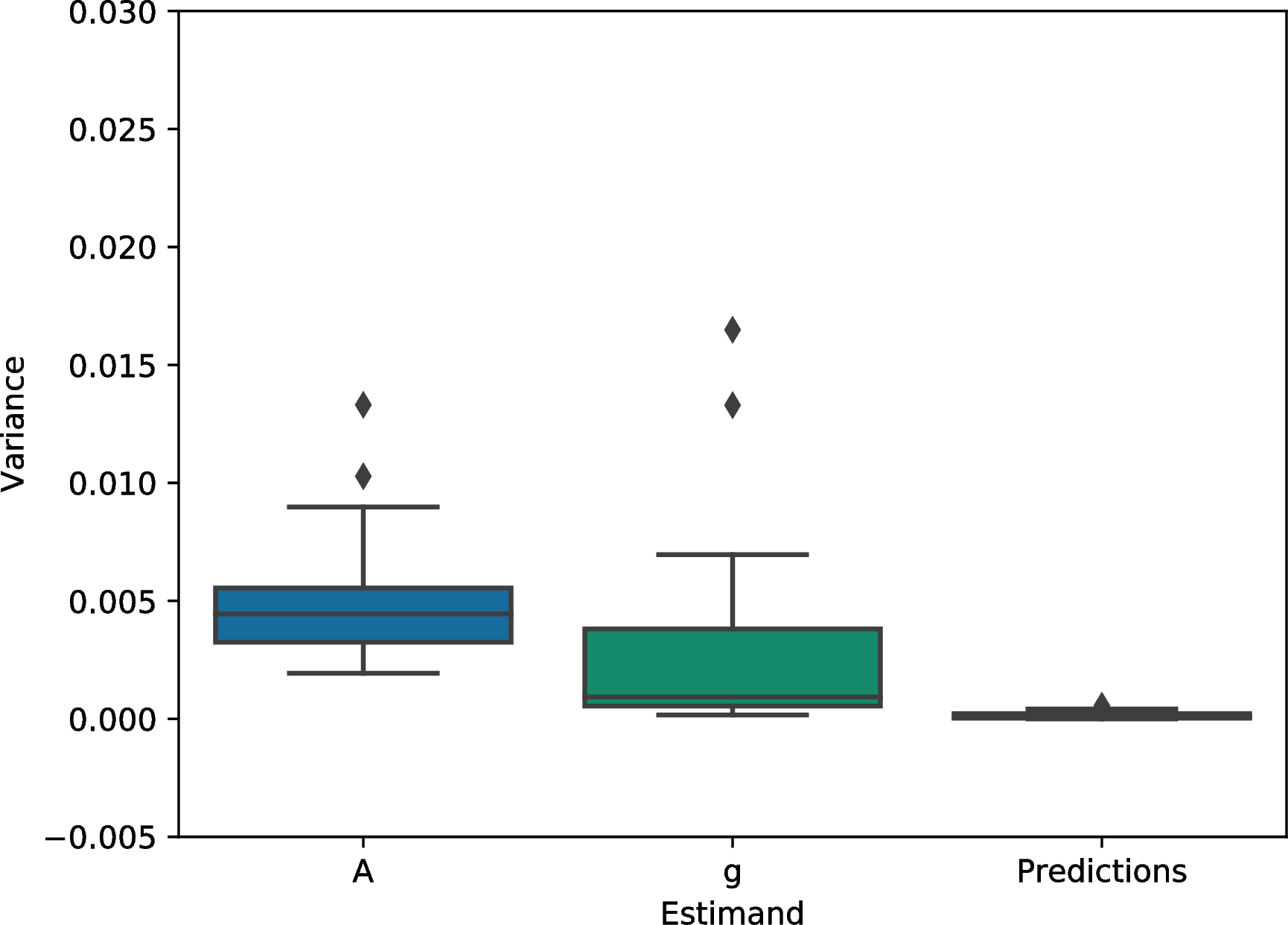

Supplement: S4 Fig — Simulated parameters were estimated once for each taxon in the denominator per simulation replicate. The variance in RMSE (y-axis) across denominators per replicate was computed to assess how choice of denominator impacted parameter estimates. (TIF) [file pcbi.1007917.s005.tif]

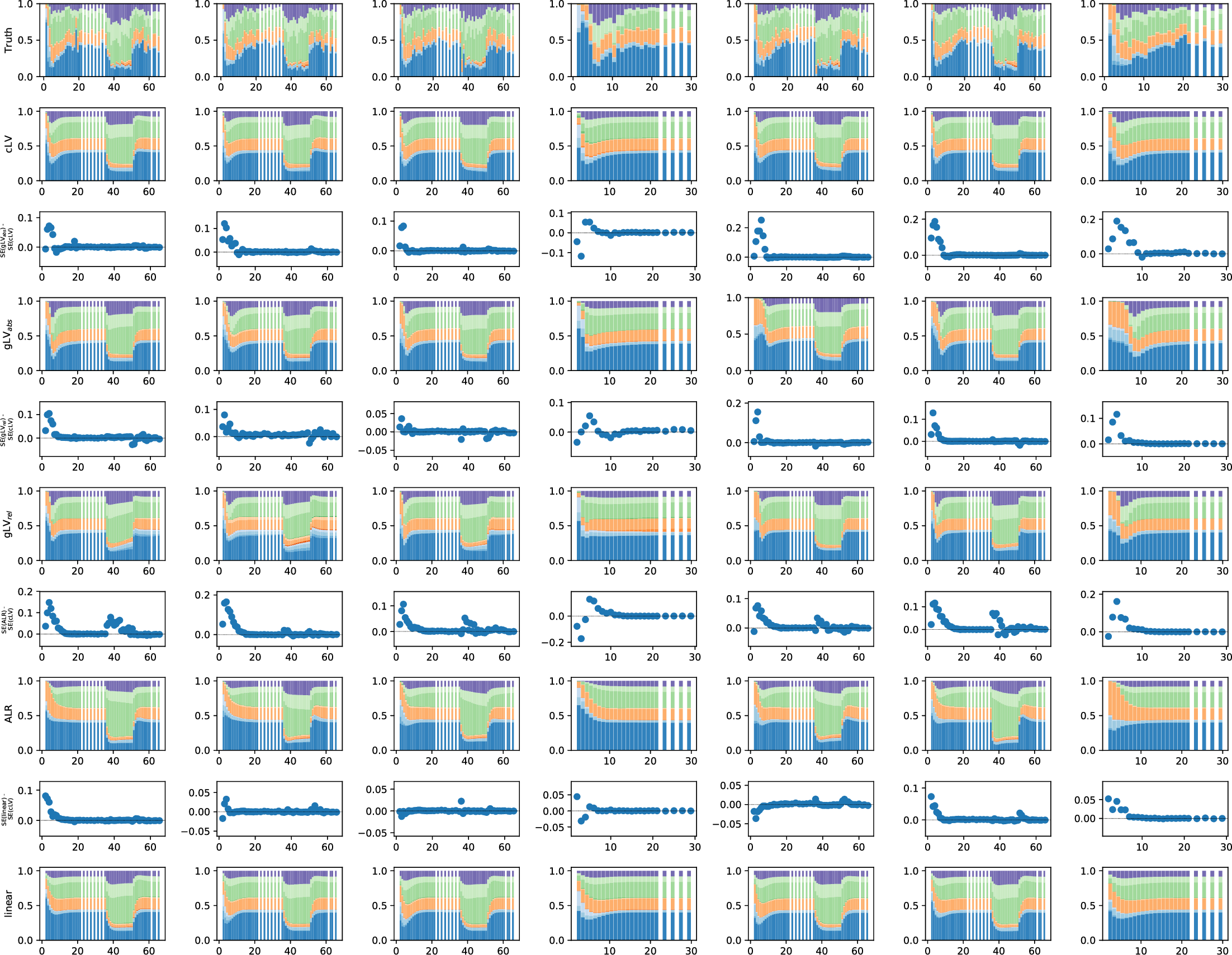

Supplement: S5 Fig — Ground truth relative abundances (top), and predicted trajectories under each model. Each column is one sample. Scatter plots give the difference in square error per time point between each model evaluated and cLV (see y-label). Values above 0 (dashed line) indicate cLV is making a better prediction, while values below zero denote the opposite. (TIF) [file pcbi.1007917.s006.tif]

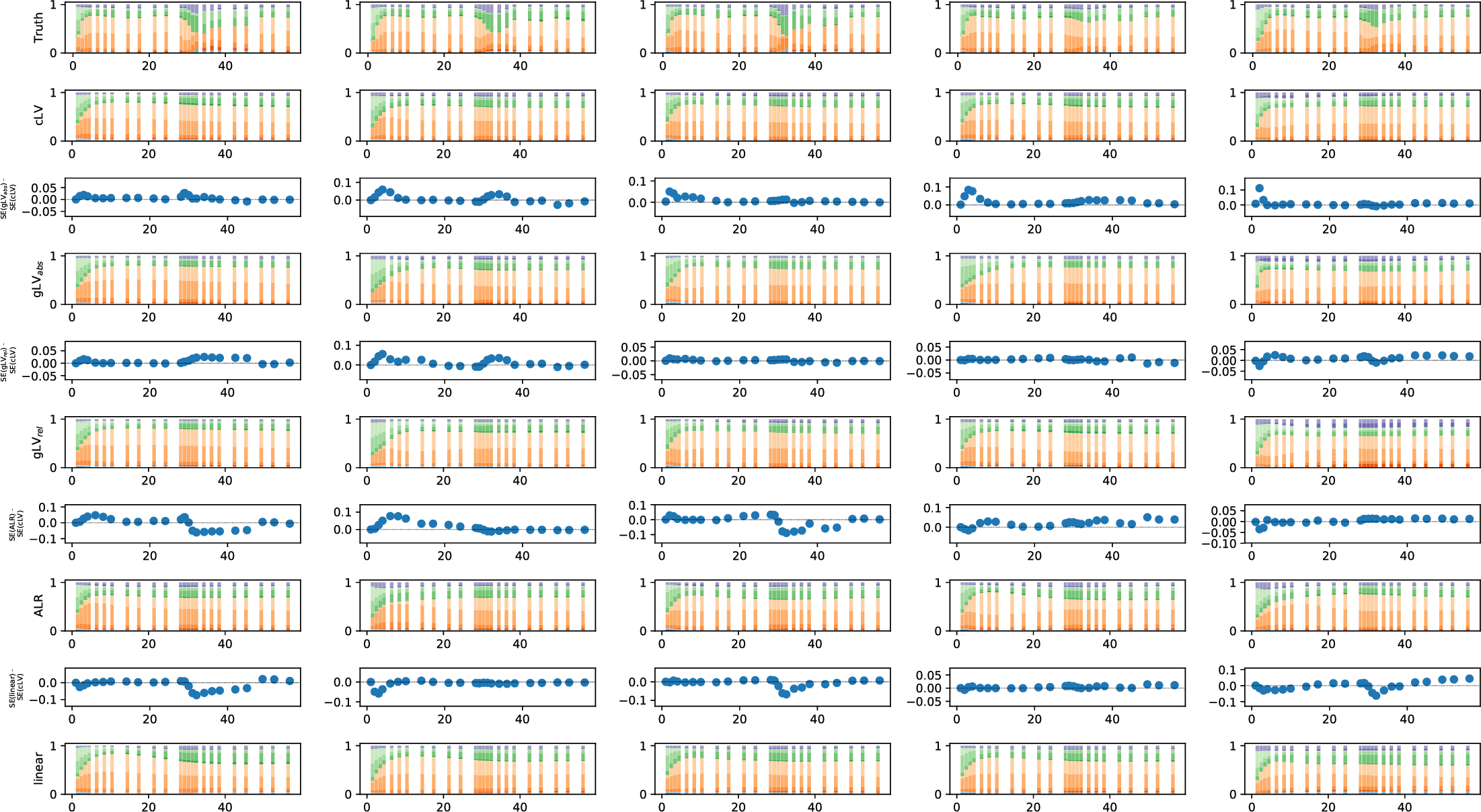

Supplement: S6 Fig — Ground truth relative abundances (top), and predicted trajectories under each model. Each column is one sample. Scatter plots give the difference in square error per time point between each model evaluated and cLV (see y-label). Values above 0 (dashed line) indicate cLV is making a better prediction, while values below zero denote the opposite. (TIF) [file pcbi.1007917.s007.tif]

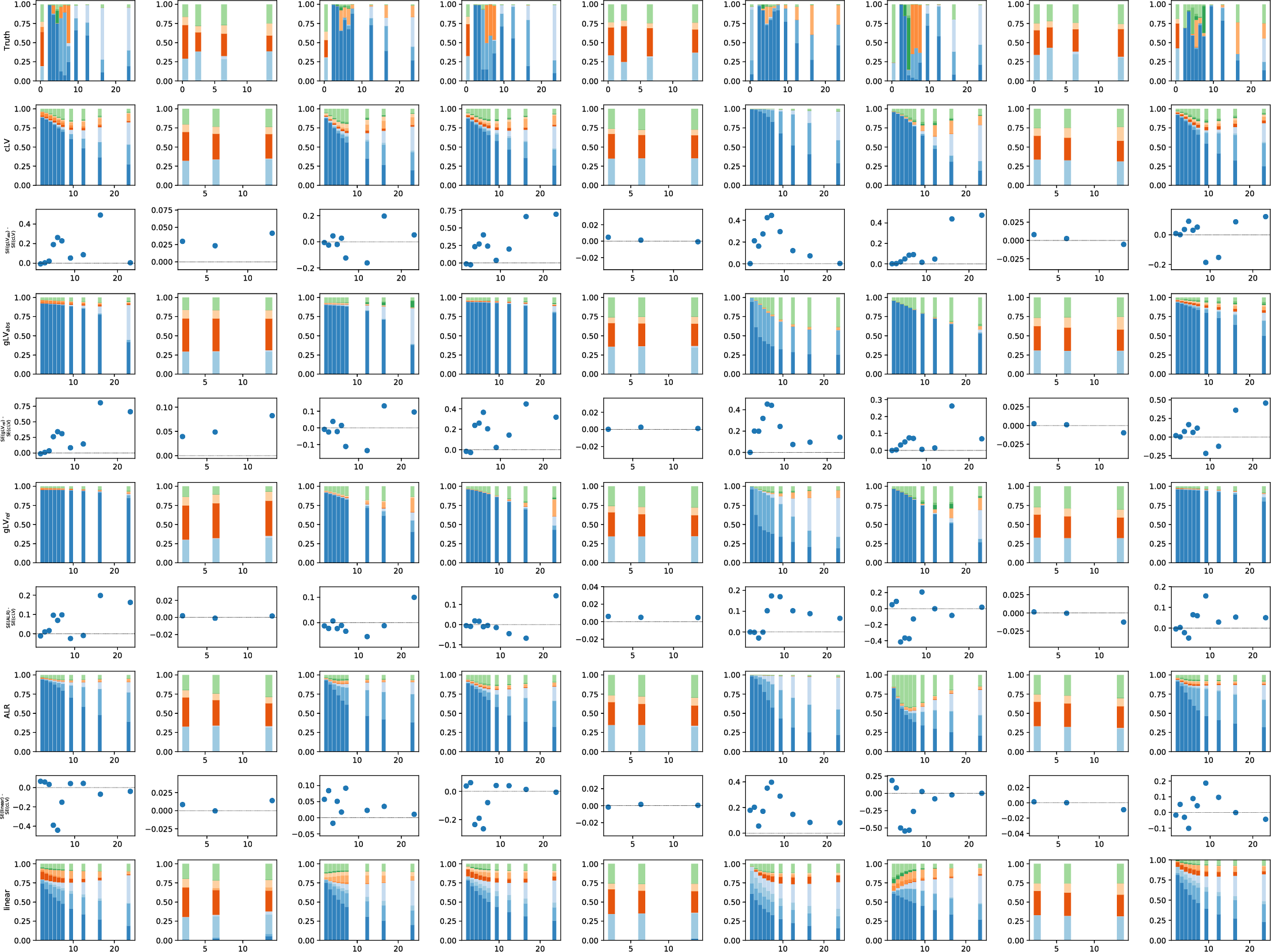

Supplement: S7 Fig — Ground truth relative abundances (top), and predicted trajectories under each model. Each column is one sample. Scatter plots give the difference in square error per time point between each model evaluated and cLV (see y-label). Values above 0 (dashed line) indicate cLV is making a better prediction, while values below zero denote the opposite. (TIF) [file pcbi.1007917.s008.tif]

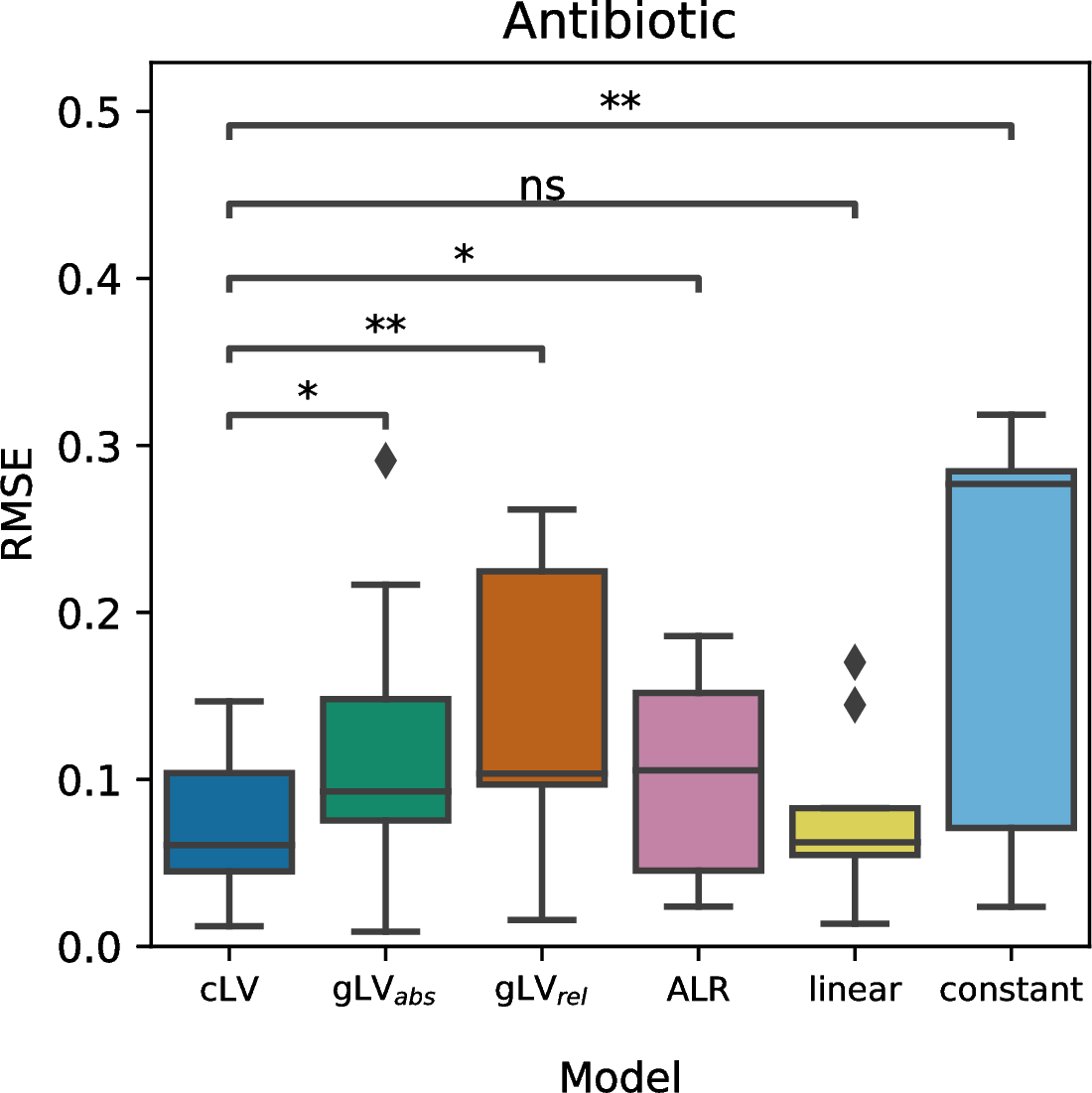

Supplement: S8 Fig — RMSE (y-axis) between ground truth and predicted final time point for each sample across models (x-axis). (TIF) [file pcbi.1007917.s009.tif]

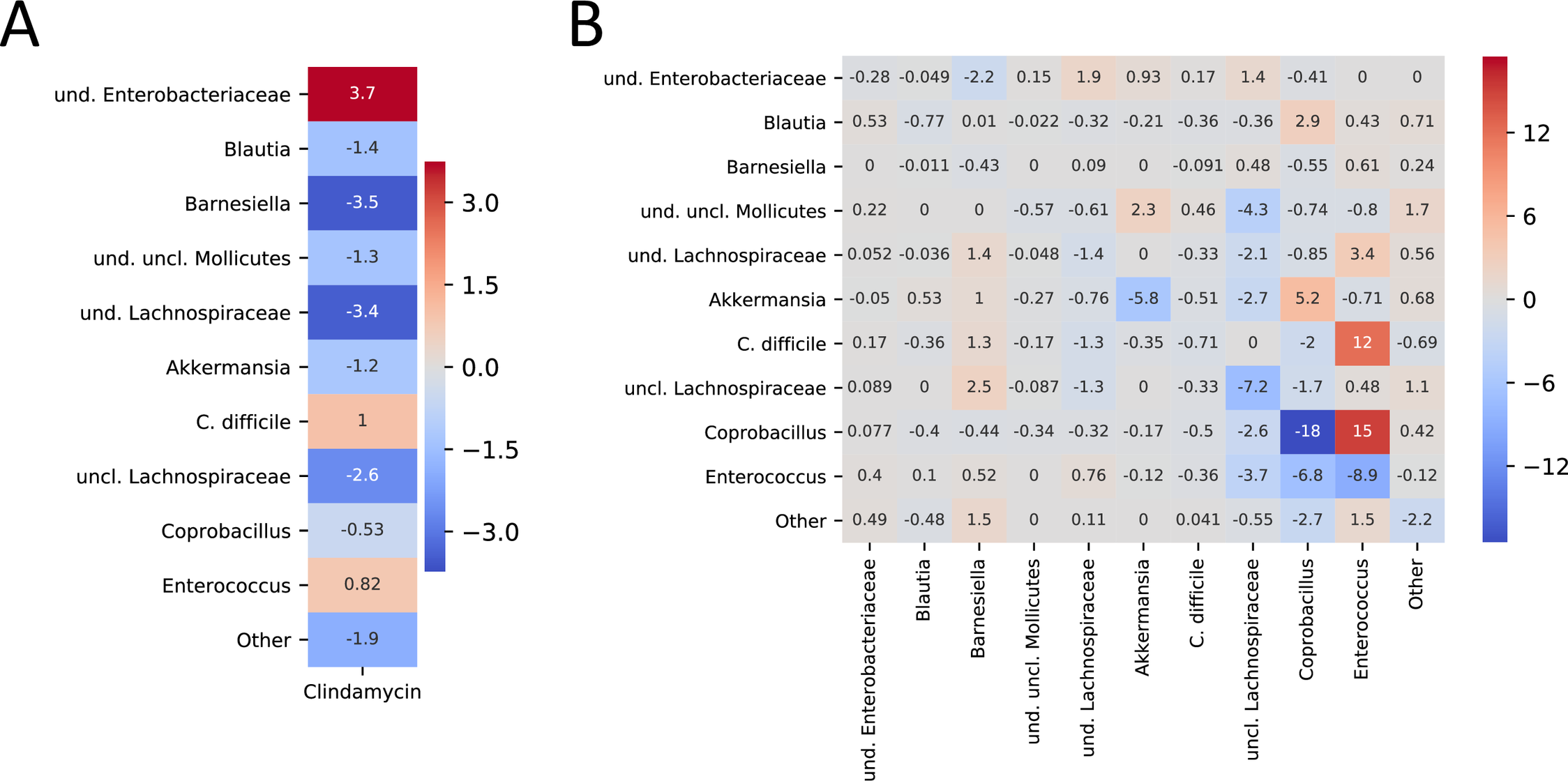

Supplement: S9 Fig — Estimated external perturbations (A) and interactions (B) using gLV with elastic net on the Antibiotic dataset. (TIF) [file pcbi.1007917.s010.tif]
