# Supplementary material for: Compositional Lotka-Volterra describes microbial dynamics in the simplex
Source: PLoS Comput Biol. 2020 May 29;16(5):e1007917. doi: 10.1371/journal.pcbi.1007917 (PMC7325845; doi:10.1371/journal.pcbi.1007917)
Supplement: Attachment — Submitted filename: Review.pdf [file pcbi.1007917.s011.pdf]

The authors present a dynamical systems model for microbiome relative abundances (compositional data.) They present two derivations of their ODE model, including one using a direct additive log-ratio transformation of compositional measurements, and which provides an interpretation relative to the standard gLV model. They then present some results on synthetic data, to justify using elastic-net regularization for parameter estimation (using a gradient-matching approach without numerical integration of the ODEs.) Next, they assess predictive performance and parameter inference of their method on four previously published datasets, with comparison to a gLV model that assumes no noise in measurements and inferred using the same elastic-net regularization approach. They additionally compare predictive performance to two linear models (either in additive log-ratio or relative abundance spaces.) Finally, they perform some analyses of a longitudinal dataset of allo-HSCT patients; they do not compare these analyses to those using other methods.

Overall, I like the work presented. The manuscript is well written, provides some nice mathematical intuition, presents generally principled and coherent analyses including on multiple real datasets, and is relevant to an important emerging area in the microbiome field, microbial ecosystem dynamics. However, I feel there are some significant issues the authors need to address before the manuscript could be published:

Major issues:

1. Although the Discussion section is quite balanced in describing limitations of the method, other parts of the manuscript don't provide this context and could be construed as misrepresenting the contributions of the work. Specific instances of this include:

    a. In the abstract, the authors state: "Specifically, deciphering how microbial species in a community interact with each other and their environment can elucidate mechanisms of disease, a problem typically investigated using tools from community ecology. Yet, such methods naively require measurements of absolute densities…" However, in the discussion the authors state a limitation of their method (and indeed compositional data in general) is that it cannot distinguish direct from indirect interactions. This is a critical goal of many studies. The authors also state that a limitation of all log-ratio based methods is the assumption that all taxa must exist at all time-points in the ecosystem, which clearly isn't biologically realistic in many settings. Moreover, as the authors later discuss, interpretability of their model is based on an assumption that the total bacterial density has low variance. In many biological settings, this isn't true. Given all these limitations of their model (and compositional data in general), there are many good reasons to try to measure absolute densities and it's not a "naïve" requirement of models!

    b. Also in the abstract, the authors state that "we show that relative abundance trajectories predicted using cLV are as accurate or better than those predicted by

gLV using absolute abundances." But, since gLV is a model of absolute abundances, it's not so surprising that it doesn't predict relative abundances as well as a model specifically developed for modeling relative abundances. I think the authors should be clearer about this being the distinction. Moreover, as I discuss later, the authors use models that assume no errors in measurements, which puts gLV at a disadvantage since it combines two separate measurements (relative abundance and total abundance), both of which are known to be noisy. I like the statement in the introduction that "Moreover, we show that cLV is as accurate as gLV in forecasting microbial trajectories in terms of relative abundances, suggesting that estimated concentrations are unnecessary for predicting community trajectories in terms of relative abundances." I think this is a much better statement of the authors' actual conclusion than what's stated in the abstract.

c. Also in the abstract, the authors state that "Our results indicate that microbial dynamics in the simplex are nonlinear, and that interactions occur in the space of relative abundances." While I agree that nonlinear microbial dynamics are likely, it's not clear that the results in this manuscript provide particularly strong evidence for that claim. On 50% of the datasets analyzed, the authors' method outperformed the alr-linear model; in the other cases there was no significant difference (and the ra-linear model slightly outperformed their model on the most complex/sparsely sampled dataset.) Moreover, while some statistically significant performance differences were seen, are these at a scale that's biologically relevant? The second part of the statement seems even more problematic that "interactions occur in the space of relative abundances." This would seem to imply some physical mechanism is in play. I think the most we can reasonably say here is that their results provide some interesting evidence of nonlinearity of dynamics in the space of relative abundances, and more datasets for comparison will be necessary to fully understand what's going on. The authors make similar statements later in the manuscript (starting on line 165) "Finally, the model suggests that dynamics are nonlinear in both the space of relative abundances and log-ratio transformed spaces." I think it's critical the authors be 100% clear what's meant here. Their MODEL is nonlinear in these spaces. However, they need to be careful about what's meant by "the dynamics." The underlying physical dynamics, or what an extension of a mathematical model (gLV) to the simplex implies? The latter is true; the former is not certain.

d. In the Discussion section starting on line 318, the authors state "This suggests that — if a researcher is interested in relative abundances alone — no usable information is gained by access to community size data. This counters intuition about more data always being better. One explanation for this discrepancy is that estimates of relative parameters (i.e. the differences between pairs of absolute parameters of gLV) are less susceptible to errors than direct biomass

estimation, perhaps because such differences cancel per-sample artifacts. Thus, the added raw data for gLV comes at a cost of noise that eliminates its marginal utility for prediction." I think this explanation is confusing and conflates several issues. I agree that total abundance as measured via qPCR is fairly noisy. But, this is all the more a reason why models and inference procedures that account for measurement noise are essential to move the field forward. It's not at all clear that if the authors compared a gLV model that included measurement noise (of both sequencing data and qPCR measurements) that their conclusion would still hold. It's quite possible the results would be different – that the added total abundance information (with its noise characteristics part of the model as well) would provide a BETTER prediction, even of relative abundances. Also, the authors are looking at datasets that all use qPCR measurements for biomass estimation. Other measurements such as spike-ins or direct cell counting may have more favorable noise characteristics. So, overall I think the authors need to be cautious about drawing very general conclusions about the utility of absolute abundance measurements from the limited models, inference procedures and datasets they've considered.

2. Section 2.2 provides some good intuition on the connection between the proposed model and standard gLV. However, it would be helpful if the authors provided some intuition on why the assumption of low variance of Var[N(t)] is essential. Can a model of dynamics over relative abundances still be defined or is essential information missing? Is the assumption more to make inference easier? Or is it to facilitate interpretation/comparison to standard gLV? Can anything be said (quantitatively) about what Var[N(t)] values will be low enough to provide a good approximation? The authors state the antibiotic dataset has a Var[N(t)] = 1.1, but it's unclear what that means. Will that scale of variation result in an accurate approximation per equation (5)? Also, with regards to alr and related transformations, can the authors address how feasible it is to find a taxon as a "reference" to use (e.g., x_D) in human datasets? In many human datasets, there may be no taxon that's consistently present across all human subjects. Realistically, how much will the smoothing assumptions as described in Section 4.6 impact such datasets? Again, I understand that some of those issues have been addressed in prior compositional literature on the microbiome, but since alr is the main transformation discussed, it's important to make some of these limitations clear upfront.

3. The section on parameter inference 2.3 seems fairly superficial and not particularly well justified. I understand the primary purpose of this section was to show that elastic-net regularization is better than other methods for this application, but I still think some more justification is needed. Also, including comparisons to OLS seems irrelevant, since prior work in the field has used ridge regression. Empirical studies presented in prior work (and theory) argue that many datasets analyzed lack sufficient information to infer parameters without regularization. So, I think comparisons should be elastic net to ridge regression, not to OLS. Regarding the synthetic data used for testing, the authors state

in the Methods (line 369) that "We choose these parameters because simulated trajectories were qualitatively similar to observed trajectories on real data." In what sense? Did they investigate a range of different parameters and similarly show robustness of their inference method? Also, the authors should provide some justification for their measurement noise model (lognormal Poisson) for simulating data; other groups have used other models including negative binomial and zero-inflated models. While I don't think the authors need to evaluate every possible noise model, since this isn't the central thrust of their work, they should at least justify why they used the particular model they did. Another issue is that elastic-net and ridge regression aren't the only possibilities for inference, and several alternate methods have been demonstrated to be superior in prior literature. These include Ba ayesian variable selection methods (Bucci et al), which has been demonstrated to outperform regularization on many of the same datasets the authors are evaluating. Again, while I understand that the primary purpose of this study wasn't to evaluate a set of inference methods, the authors should be clearer about the limited scope of their investigations into parameter inference for their model.

4. In analyses of the real datasets, there are several issues.

    a. The authors state that their method performs best when variance of the total abundance is lower, which is theoretically justified based on their modeling assumptions. They later state in the Discussion that total abundance measurements may be quite noisy. Both these factors were recognized in Bucci et al's work (Gen Biol 2016) and they performed an analysis testing whether an assumption of constant biomass would change inferences and predictions, indeed on two of the same datasets analyzed in the present manuscript (Diet and C. diff.) The conclusion of Bucci et al was that the assumption of constant biomass was least justifiable for the C. diff dataset. Given all these findings, and if the authors' main question is prediction of relative abundances, would assuming constant biomass in the gLV model indeed lead to more accurate prediction of RELATIVE ABUNDANCES than using the biomass data? Since the authors' assume measurements are noise-free in their model, this seems important to at least try.

    b. As I understand it, C. diff is being treated as an on-off perturbation. Why? It's clearly a time-varying change in the ecosystem. The previous work from which the present authors obtained the data treated C. diff as another organism in the ecosystem that was introduced later in the time-course. This would seem to make most sense and could well explain why "Both models performed similarly on the C. diff dataset, but neither captured a community disturbance due to the introduction of C. diff." (starting on line 232.) This is discussed somewhat in the Discussion section, but it seems a bit odd to analyze a data set using a method that can't capture a central feature of the biological system under study (invasion of a pathogen.) In the Discussion section the authors state that "A

further fundamental limitation of all models based on log-ratios is the inability to describe extinction and colonization. Each taxon is assumed to exist at each time point." Can the authors elaborate on this? It's not clear to me why this is true, since they use a smoothing method that effectively replaces zeros in the data. Is the inability to describe extinction and colonization an absolute limitation of log-ratio based models or just an issue of less accuracy? Also, in the Methods section the authors describe excluding 6 taxa from their analyses. This wasn't done in the original Bucci et al work. Why did the authors need to do so here? Did it have to do with their not having a noise-model and therefore not being able to handle lower abundance species? Could excluding all these organisms (~50% of the community) also account for their not being able to see a "community disturbance due to the introduction of C. diff?"'

a. For the allo-HSCT patient data, the authors don't compare performance of their method with the others on the Enteroccocus prediction task. Would the linear methods or gVL (with assumption of constant biomass) yield similar AUCs and/or interpretable interactions? Since the theme of the paper seems to be comparisons between these methods, comparing performance of the other methods on the same task seems relevant. Also, I'm not sure what's meant by a strong positive effect of Lachnoclostridium on Enterococcus and Bacteroides on Enterococcus. Since the model can't distinguish direct from indirect effects, as stated in the Discussion section, how are these effects to be interpreted?

Minor issues or comments:

Starting on line 50: "However, gLV-based models describe dynamics in terms of absolute densities of taxa and require measurements of community size—either from quantitative PCR or spiked-in samples of known concentrations—in addition to sequencing counts of constituent taxa (Cao et al., 2017)."

FACS or other cell-counting methods are another possible way to estimate the total community size, and should be mentioned.

Starting on line 67 "Sequencing counts only contain information about the relative abundances of community members: the total number of sequencing reads is independent of the size of the community, and relative abundances only provide information about how the proportions of each species change over time."

It's true that the total number of sequencing reads is independent of the size of the community. However, it's also important to stress that sequencing reads are what's actually being measured, NOT relative abundances. Indeed, sequencing reads introduce an additional layer of variability that need to be accounted for to properly model the data.

Regarding the quantile method of Shenhav et al (2019) (line 74), for a sufficiently large number of bins the interaction structure would be preserved. Is the problem that adaptive binning is a difficult problem in this setting?

The interpretation discussed starting on line 152 is interesting. Equation 6 implies higher order interactions than gLV, i.e., terms involving products of \pi_i, \pi_j, and \pi_k. Moreover, for the gradient-matching approach to parameter estimation for these ODEs, for gLV, inference of the coefficients for taxon i is "local" in the sense that it involves only g_i, A_i, and B_i. With the cLV model and this inference method, the g, A, and B coefficients for other taxa directly effect dpi_i/dt. This would seem to have implications for the runtime of the inference algorithm…?

In line 156, the authors state "More generally, cLV provides intuition on how to model relative abundances dynamic even when the approximation does not hold."

I agree with this, but I think it'd be helpful for most readers if the authors could be explicit about that intuition here.
