# Supplementary material for: Compositional Lotka-Volterra describes microbial dynamics in the simplex
Source: PLoS Comput Biol. 2020 May 29;16(5):e1007917. doi: 10.1371/journal.pcbi.1007917 (PMC7325845; doi:10.1371/journal.pcbi.1007917)
Supplement: Attachment — Submitted filename: Response Only.pdf [file pcbi.1007917.s013.pdf]

[1.1]
Reviewer #1: The authors have done a good job of responding to the technical queries of the reviewers. The paper remains a solid - if somewhat esoteric - contribution to the literature and I have no further suggestions for improvements.

[1.1]
We thank the reviewer for their comments.

Reviewer #2: The authors have done substantial work to improve the manuscript and have addressed all of my major concerns. In particular, I really appreciate the authors' work deriving new qualitative and quantitative understanding of what can be inferred by their model (e.g., the new Section 2.6). The microbiome field really needs this type of careful and thoughtful analysis to get beyond blind application of generic statistical and machine learning methods.

At this point, my critique involves changes for clarity and consistency in the manuscript.

Also, I'd like to apologize for the amount of time it took me to re-review. I received the manuscript while on a tight grant deadline and informed the editorial office I wouldn't be able to re-review until the first week in March. However, due to the COVID-19 situation, both personally and events at my institution, I've been delayed in getting this done.

Comments:

[2.1]
Pg 2, starting line 35: "We show that relative abundances are sufficient to learn the process governing microbial dynamics…"
I would cut this sentence or revise. As written, this sentence could be misinterpreted as: 1) you're learning the actual physical process (rather than a model) and, 2) absolute abundance measurements provide no useful information.

[2.1]
Now pg 3, starting line 45. We have revised this and the following sentences to more precisely describe our results. It now reads:

"Across three real datasets, we show that relative abundances are sufficient to describe compositional dynamics. Additionally, we show that models trained on relative abundances alone predict future compositions as well models trained on absolute abundances. Finally, we provide criteria for when direct effects, which typically can only be learned from absolute abundances, are recoverable for relative data."

We have replaced "process governing microbial dynamics" with "describe compositional dynamics," which more accurately characterizes our results. Furthermore, the final sentence implies that, when only relative abundances are considered, information is lost when compared to absolute measurements.

[2.2]
Pg 4, line 79: "Yet, binning taxa into quantiles loses fine-grained information…"
I would soften this to "MAY lose fine-grained information…"

This depends on the discretization scheme and what the model is trying to learn. For relationships such as signs of interactions, discretization approaches could potentially work quite well.

[2.2]
Now pg 4, line 94. We have made this change.

[2.3]
Pg 5, line 107: "…recapitulate a mechanism of C. difficle [typo] colonization…"
I wouldn't really say the cited work offers a mechanism of C. difficile colonization. Maybe clearer to call it a "proposed directed microbe-microbe interaction network with C. difficile" or something similar, since that's what you're ultimately comparing to. This same phrase is used elsewhere in the manuscript, so should be changed throughout.

[2.3]
Now pg 6, line 131. This now reads "proposed interaction network with C. difficile inferred using absolute densities."

We have made similar changes to the manuscript in the following locations:
1. Pg 17, line 386
2. Pg 19, line 450

[2.4]
Pg 8, line 166: This is a really nice insight! It allows one to easily see what the "compositional" contribution is in these types of models.

[2.4]
We thank the reviewer for the comment!

[2.5]
Pg 9, line 186: "…form of equation (6) makes direct application of these methods challenging…"
I would revise to "…form of our model (e.g., equation 6) does not allow us to readily apply these methods…"
The model in Bucci et al is fully Bayesian and hierarchical. It's not clear to me how the cLV model could best be recast as a fully Bayesian model (or that equation 6 is the only issue) and what inference method would be most efficient. That's an interesting question for future research!

[2.5]
Now pg 10, line 227. We have made this change.

[2.6]
Pg 9, line 207: "…we wanted to ensure that difference [typo] choices of denominator do not affect quality of inference."
This is a very nice result that demonstrates robustness of the method.

[2.6]
Now pg 11, line 255: difference => different.

Computer Science Building Mail Code 0401 500 W 120th St New York, NY 10027 212-939-7000 Fax 212-666-0140

[2.7]
Pg 10, line 228: "…"Diet" dataset included 7 mice. 5 mice were fed from a high-fiber diet…"
Just to clarify, this dataset only contains absolute abundances. The concentrations of the individual taxa were measured via qPCR. Relative abundances weren't measured in these experiments.

[2.7]
Now pg 12, line 287. We have added a sentence to clarify this point:

"The *C. diff* dataset and Antibiotic dataset combined 16S sequencing with qPCR to estimate relative abundances and community size separately, while the Diet dataset used qPCR for individual taxa to measure concentrations."

[2.8]
Pg 10, line 238: "For this particular task, we chose to use ridge regression since elastic net may choose to zero out different parameters for each model, making direct comparison challenging."
I'm not sure what's meant here or why ridge regression was used here and elastic net was used elsewhere. Both ridge regression and elastic net are shrinkage estimators that don't zero out regression coefficients (whereas the lasso algorithm does), but just bias them to small values. So, why won't elastic net work just as well here? Based on your analyses on simulated data, it doesn't seem there's a meaningful difference in performance between the two methods. So, couldn't you just use ridge regression throughout the manuscript for consistency? Or, are you using some version of elastic net that actually does zero out parameters?

[2.8]
Elastic net regularization uses a linear combination of the L2 penalty for ridge regression with the L1 penalty for lasso. Therefore, ridge regression and lasso are special cases of elastic net. The L1 penalty encourages sparsity, while the L2 penalty performs shrinkage.

Elastic net was developed to overcome a limitation of the lasso, where the lasso would only select one of a set of correlated predictors. Elastic net, in contrast, will include or exclude the entire set of parameters. This means that elastic net does zero out parameters like lasso (see Zou and Hastie 2005 in the references for more details).

In comparing model parameters, we wanted to maximize the number of comparisons performed. This means removing the L1 penalty that encourages model sparsity, and hence using ridge regression.

The main reason we choose elastic net over ridge regression for prediction: we could include regularization parameters corresponding to ridge regression in the cross validation procedure.

[2.9]
Pg 11, line 248: "Nonetheless, correspondence between interactions and external effects was strong among the three datasets we explored."
This is another nice result that empirically confirms when compositional effects may be important and when they're not.

[2.9]
We thank the reviewer for the comment!

[2.10]
Pg 12, line 262: "To avoid overfitting, which would cause models with more parameters to perform better, we only evaluated predicted trajectories on held out test data using leave-one-out cross validation. That is, for each dataset we held out one sample at time, and trained models on the remaining data."
As written, I think this is a bit confusing as to the reason why you're doing cross-validation. Maybe something like: "We evaluated models based on a measure of generalization performance, or the ability to predict unseen data. Generalization performance metrics inherently penalizes models that overfit, or use parameters to fit noise in data rather than model actual signal. These metrics allow for principled comparison of models with different structures or numbers of parameters. In our case, we used a metric that evaluated predicted trajectories on held out test data via leave-one-out cross validation. That is, for each dataset we held out one time-series in turn, and trained models on the remaining data."

[2.10]
Now pg 13, line 324. We have made this change. We thank the reviewer for the suggestion.

[2.11]
Pg 13, line 284: "Second, none of the models captured a community disturbance resulting from the introduction of C. difficle [typo]."
I continue to find this statement confusing. What is meant by the models not capturing a community disturbance? Since you're modeling C. diff as one of the microbes in the ecosystem, doesn't that capture the disturbance? I think more clearly defining what's meant by a community disturbance (in terms of your model and this particular dataset) is needed.

[2.11]
Now pg 15, line 352. This sentence now reads: "Second, none of the models captured a fluctuation in community composition, where the community briefly moved away from stability, in the 5 time points immediately after introduction of *C. difficile*."

[2.12]
Pg 13, line 292: "…suggested criteria for when an absolute term…"
I'd explicitly state "absolute growth rates and interaction terms" since it's unclear what "term" means until later in the paragraph.

[2.12]
Now pg 15, line 361. We have made this change.

[2.13]
Pg 13, line 298: I don't entirely follow the logic that the probability of getting the sign correct is 75%. It's not clear to me that the assumption of a symmetric distribution around zero implies equal probability of the four cases. Doesn't it depend on P(|A_{ij}| > |A_{D_{j}}|)?

[2.13]
Now pg 16, starting on line 362. The reviewer is correct, the quantity depends on P(|A_{ij}| > |A_{Dj}|). We have revised this section, removing this particular argument from the manuscript.

[2.14]
Pg 14, line 305: This paragraph is overall a bit confusing. Perhaps the term "optimal" rather than "right" would be better. Also, the statement that "…these do not need to be the same denominator…" is confusing. I think some copyediting will help here.

[2.14]
Now pg 16, line 362. We have revised all of the "Interpreting model parameters" section for clarity.

[2.15]
Pg 15, line 347: "Notably, cLV more accurately forecast community trajectories than gLV across all three datasets we explored."
Be clear that the forecasting task is relative abundances, i.e., "Notably, cLV more accurately forecast community trajectories of relative abundances than gLV across all three datasets we explored."

[2.15]
Now pg 18 line 430. We have changed this sentence to "Notably, cLV more accurately forecast *relative abundances* than gLV…"

[2.16]
Pg 15, line 351: "One explanation for the discrepancy is that gLV is penalized twice for noisy data, while cLV only once."
I disagree that this is an inherent problem with the gLV equations. It relates more to the statistical noise model (or lack thereof.) You're comparing two models that lack explicit noise models. Models using either gLV or cLV dynamics could use explicit noise models. This has already been shown to benefit gLV-based models. It would likely benefit a cLV model as well. The issue as to whether absolute abundances can be accurately measured is a separate point. There are technical challenges to making these measurements and the first methods employed were fairly poor. But, there have already been and continue to be improvements in the experimental methodologies.

I think a more interesting and relevant question from the computational perspective (and for purposes of this manuscript) is why gLV underperforms when it's given only relative abundance information. As you've pointed out, this makes an assumption of constant biomass, which is clearly wrong in many cases. Again, as you've pointed out, gLV isn't a model for compositional data. So, unless there's constant biomass, it's the wrong model for relative abundances. This is the most important take-away for readers. The intricacies of experimental technologies and statistical error models are secondary issues.

[2.16]
Now pg 18, line 430. We agree with the reviewer, and have revised the paragraph accordingly.

[2.17]
Pg 16, Line 372: "…our contribution is a formulation of when parameters can be recovered mathematically…"
This is a bit hard to follow. Maybe something like "..our contribution is a mathematical formulation of criteria for when absolute parameters can be recovered from relative abundance information."

[2.17]

Now pg 19, line 454. We have made this change.

[2.19]
Regarding the C. diff dataset in Bucci et al:
Just to be clear, because this was a gnotobiotic experiment, we knew exactly which taxa were introduced, and from the sequencing data, we did not detect any contaminants. Any additional OTUs beyond the taxa actually present are bioinformatic artifacts and occur at very low abundances. So, in your analyses, it makes most sense to include all of the taxa experimentally introduced and exclude any bioinformatic artifacts, as done in the original MDSINE analyses. The MDSINE analyses were done using older bioinformatics pipelines. In recent gnotobiotic experiments we've done with the same taxa, we can recover nearly exactly the same ASVs/OTUs as there are actual taxa.

[2.19]
Indeed, this is our understanding as well. We are using MDSINE, as in the original analyses as the comment advocates, to obtain denoised concentrations for *C. diff* dataset for downstream analysis.

[2.20]
General: there are a number of typos throughout, particularly in the new sections. Careful copyediting is needed.

[2.20]
We have revised our manuscript for spelling, grammar, and clarity.

Computer Science Building Mail Code 0401 500 W 120th St New York, NY 10027 212-939-7000 Fax 212-666-0140
