# Supplementary material for: Compositional Lotka-Volterra describes microbial dynamics in the simplex
Source: PLoS Comput Biol. 2020 May 29;16(5):e1007917. doi: 10.1371/journal.pcbi.1007917 (PMC7325845; doi:10.1371/journal.pcbi.1007917)
Supplement: Attachment — Submitted filename: Response to Reviewers.docx [file pcbi.1007917.s012.docx]

Department of Computer Science

Columbia University

500 W 120^th^  St., New York

NY 10027-7003

email: itsik@cs.columbia.edu

Tel: +1-212-939-7135

January 24, 2020

Dear Dr. Dakos and Dr. Komarova,

Thank you for considering our revised manuscript “Compositional Lotka-Volterra describes microbial dynamics in the simplex.” We are grateful for your and the reviewer’s thoughtful comments.

In addition to the changes discussed in the Cover Letter, please find attached our point-by-point response below. Reviewer’s comments are bold, and our response is plain text. We hope you will find our responses satisfactory.

Sincerely,

Tyler Joseph	Itsik Pe’er

**Reviewer #1: In this manuscript, Tyler A. Joseph and colleagues adopt ecology models of community dynamics to a compositional space. The paper is clearly written, and the topic will likely be of at least modest interest to the community of researchers interested in the theory of how to apply ecological principles to sequence count data. It is less clear that the progress in theory the paper represents will be of immediate use to the larger community of researchers who have longitudinal 16S or WGS-metagenome data and are searching for ways to relate these data to clinical parameters.**

**[1.1]**

**For table #1, could the authors add columns indicating the total sample size for each study, the # of free parameters in each corresponding model and the method used for generating absolute abundance in each study. In general, the authors could be more explicit about the relationship between the number of free parameters in the models they used and the sample size. It is unclear from the current version of the manuscript if the models tend to have nearly as many or more parameters than the number of samples. In such cases, good fits to the data are not hard to achieve (as the physicist von Neumann was alleged to have said, “With four parameters I can fit an elephant, and with five I can make him wiggle his trunk.”). Some consideration of model flexibility given model complexity should be added to the discussion.**

[1.1] In Table 1, we now list the total number of observed time points in addition sample size and data collection methods. We have additionally added a table (Table 2) to the model comparison (section 2.5) with the number of free parameters of each model.

We appreciate the reviewer’s concern about overfitting. Indeed, in our model comparison we follow standard procedures from the machine learning field to evaluate each model. Importantly, this means each model is evaluated on data not used for parameter inference. This avoids problems of overfitting, where models with more parameters will perform better.

In addition, we highlight this concern in the text. We have included text in section 2.3 (lines 181-183) motivating use of regularization in models that fit more parameters than samples. We discuss this issue again, and how we avoid it, in section 2.5 (lines 262-265).

**[1.2]**

**Do the cLV and gLV and linear models (equations (11) and (12)) all have the same number of free parameters? Or can the modest increases in performance seen by cLV in fitting the data be explained by an increase in the number of free parameters?**

[1.2] gLV has more free parameters than cLV, so this does not explain the increase in performance. We also evaluated each model on unseen test data to avoid overfitting.

**[1.3]**

**The glv equations are not universally appreciated as being appropriate for ecological modeling and some discussion of this should be added to the introduction or discussion (for example J. theor. Biol. (1989) 139, 311-326; Coupling in Predator-Prey Dynamics: Ratio-Dependence; ROGER ARDITIt AND LEV R. G1NZBURG).**

[1.3] We thank the reviewer for this comment. We agree that this is important to highlight in our study. We have added a paragraph in the Discussion on the limitations of gLV (lines 385-391).

**[1.4]**

**The section on “clinical relevance” is not particularly compelling. It’s not immediately clear what an ROC based on “the greatest predicted fraction of Enterococcus (across all predictions) as the prediction threshold” could mean. This seems a bit circular as the model is apparently being used to measure its own predictions. This section (section 2.6) could potentially be removed in its entirety. Alternatively, the authors could find a more traditional clinical prediction (case/control status, probability of getting an infection) or some other feature more clearly related to a patient health outcome as a test of their model. They could also more directly demonstrate the utility of their model by directly comparing to more frequently used data analysis schemes (for example, how does their model do when compared to a random forest classifier built with leave X out classification).**

[1.4] On further inspection, we agree with the Reviewer’s assessment on the importance of section 2.6. As a result, we have replaced it with a new section (also section 2.6) on interpreting model parameters in relation to the choice of denominator.

As we noted in the previous manuscript, the parameters of cLV correspond to differences in parameters of gLV. We provided empirical evidence for this claim in section 2.4. One consequence is that cLV provides criteria for when an absolute interaction may be recoverable from relative data, given a particular choice of denominator. We provide evidence for this claim in the new section 2.6.

We believe this new section better fits with the motivation discussed under the revised bullet points at the end of section 2.2.

**[1.5]**

**Could the authors expand their discussion of the choice of the denominator taxa in their method? From the methods section, different strategies were taken for choosing the denominator in the different datasets. Could the authors demonstrate that the superiority of the cLV method was robust to these choices. That is to say, if a different choice for the denominator was used, does cLV still out-perform the other methods?**

[1.5] We now provide motivation for a particular choice of denominator, leading to a quantitative way to choose the denominator for each dataset described in the Methods (section 4.6). In addition, we have performed simulations that demonstrate cLV is robust to choice of denominator both in accuracy of parameter estimates and predicted trajectories (lines 193-194, lines 206-216). We detailed these new results in section 2.3.

**Reviewer #2. The authors present a dynamical systems model for microbiome relative abundances (compositional data.) They present two derivations of their ODE model, including one using a direct additive log-ratio transformation of compositional measurements, and which provides an interpretation relative to the standard gLV model. They then present some results on synthetic data, to justify using elastic-net regularization for parameter estimation (using a gradient- matching approach without numerical integration of the ODEs.) Next, they assess predictive performance and parameter inference of their method on four previously published datasets, with comparison to a gLV model that assumes no noise in measurements and inferred using the same elastic-net regularization approach. They additionally compare predictive performance to two linear models (either in additive log-ratio or relative abundance spaces.) Finally, they perform some analyses of a longitudinal dataset of allo-HSCT patients; they do not compare these analyses to those using other methods.**

**Overall, I like the work presented. The manuscript is well written, provides some nice mathematical intuition, presents generally principled and coherent analyses including on multiple real datasets, and is relevant to an important emerging area in the microbiome field, microbial ecosystem dynamics. However, I feel there are some significant issues the authors need to address before the manuscript could be published:**

We thank the reviewer for their thorough and thoughtful comments on our manuscript.

**Major issues:**

1. **Although the Discussion section is quite balanced in describing limitations of the method, other parts of the manuscript don’t provide this context and could be construed as misrepresenting the contributions of the work. Specific instances of this include:**

[2.1] We agree with the reviewer’s assessment. We have carefully rewritten the manuscript to better distinguish claims supported by the data from speculation requiring future work. In addition, we more careful to highlight limitations of compositional data compared with absolute data. In particular, we have included a new section (new section 2.6) investigating when direct effects --- typically only estimable from absolute measurements -- are inferable from compositional data. We note that the majority of changes to the manuscript are in the Results and Discussion, though we have also rewritten parts of the Abstract and Introduction with this in mind.

**[2.1.a]**

**In the abstract, the authors state: “Specifically, deciphering how microbial species in a community interact with each other and their environment can elucidate mechanisms of disease, a problem typically investigated using tools from community ecology. Yet, such methods naively require measurements of absolute densities...” However, in the discussion the authors state a limitation of their method (and indeed compositional data in general) is that it cannot distinguish direct from indirect interactions. This is a critical goal of many studies. The authors also state that a limitation of all log-ratio based methods is the assumption that all taxa must exist at all time-points in the ecosystem, which clearly isn’t biologically realistic in many settings. Moreover, as the authors later discuss, interpretability of their model is based on an assumption that the total bacterial density has low variance. In many biological settings, this isn’t true. Given all these limitations of their model (and compositional data in general), there are many good reasons to try to measure absolute densities and it’s not a “naïve” requirement of models!**

[2.1.a] The reviewer is correct. Measuring absolute densities is not a naive requirement of models, and we have removed this term from the Abstract.

**[2.1.b]**

**Also in the abstract, the authors state that “we show that relative abundance trajectories predicted using cLV are as accurate or better than those predicted by gLV using absolute abundances.” But, since gLV is a model of absolute abundances, it’s not so surprising that it doesn’t predict relative abundances as well as a model specifically developed for modeling relative abundances. I think the authors should be clearer about this being the distinction. Moreover, as I discuss later, the authors use models that assume no errors in measurements, which puts gLV at a disadvantage since it combines two separate measurements (relative abundance and total abundance), both of which are known to be noisy. I like the statement in the introduction that “Moreover, we show that cLV is as accurate as gLV in forecasting microbial trajectories in terms of relative abundances, suggesting that estimated concentrations are unnecessary for predicting community trajectories in terms of relative abundances.” I think this is a much better statement of the authors’ actual conclusion than what’s stated in the abstract.**

[2.1.b] We have changed this sentence in the Abstract to use the same language in the Introduction. It now reads: “we show that cLV is as accurate as gLV in forecasting microbial trajectories in terms of relative abundances.”

**[2.1.c]**

**Also in the abstract, the authors state that “Our results indicate that microbial dynamics in the simplex are nonlinear, and that interactions occur in the space of relative abundances.” While I agree that nonlinear microbial dynamics are likely, it’s not clear that the results in this manuscript provide particularly strong evidence for that claim. On 50% of the datasets analyzed, the authors’ method outperformed the alr-linear model; in the other cases there was no significant difference (and the ra-linear model slightly outperformed their model on the most complex/sparsely sampled dataset.) Moreover, while some statistically significant performance differences were seen, are these at a scale that’s biologically relevant? The second part of the statement seems even more problematic that “interactions occur in the space of relative abundances.” This would seem to imply some physical mechanism is in play. I think the most we can reasonably say here is that their results provide some interesting evidence of nonlinearity of dynamics in the space of relative abundances, and more datasets for comparison will be necessary to fully understand what’s going on. The authors make similar statements later in the manuscript (starting on line 165) “Finally, the model suggests that dynamics are nonlinear in both the space of relative abundances and log-ratio transformed spaces.” I think it’s critical the authors be 100% clear what’s meant here. Their MODEL is nonlinear in these spaces. However, they need to be careful about what’s meant by “the dynamics.” The underlying physical dynamics, or what an extension of a mathematical model (gLV) to the simplex implies? The latter is true; the former is not certain.**

[2.1.c] We have removed this sentence from the Abstract. We clarified other instances to highlight the potential benefits of nonlinear *models* over linear *models*. In particular, we have revised the Discussion section comparing cLV to the additive log-ratio model and linear model on relative abundances with this in mind (lines 360-367).

**[2.1.d]**

**In the Discussion section starting on line 318, the authors state “This suggests that — if a researcher is interested in relative abundances alone — no usable information is gained by access to community size data. This counters intuition about more data always being better. One explanation for this discrepancy is that estimates of relative parameters (i.e. the differences between pairs of absolute parameters of gLV) are less susceptible to errors than direct biomass estimation, perhaps because such differences cancel per-sample artifacts. Thus, the added raw data for gLV comes at a cost of noise that eliminates its marginal utility for prediction.” I think this explanation is confusing and conflates several issues. I agree that total abundance as measured via qPCR is fairly noisy. But, this is all the more a reason why models and inference procedures that account for measurement noise are essential to move the field forward. It’s not at all clear that if the authors compared a gLV model that included measurement noise (of both sequencing data and qPCR measurements) that their conclusion would still hold. It’s quite possible the results would be different – that the added total abundance information (with its noise characteristics part of the model as well) would provide a BETTER prediction, even of relative abundances. Also, the authors are looking at datasets that all use qPCR measurements for biomass estimation. Other measurements such as spike-ins or direct cell counting may have more favorable noise characteristics. So, overall I think the authors need to be cautious about drawing very general conclusions about the utility of absolute abundance measurements from the limited models, inference procedures and datasets they’ve considered.**

[2.1.d.] The reviewer is correct, and we have rewritten the discussion to highlight these concerns and be more careful in our conclusions (lines 347-359). In particular, we emphasize that gLV is penalized twice for noisy data: once from estimating biomass estimation and once from estimating relative abundances. We have also performed several steps in our analysis to attempt to mitigate problems stemming from sequencing noise.

On the *C. diff* dataset we now use the denoising step from MDSINE to estimate bacterial concentrations. We were unable to perform this step for the Diet or Antibiotic datasets. Inspecting the code from MDSINE, it appears that estimated concentrations from the Diet dataset are used directly for downstream inference, likely because this dataset used a different data collection method. We would very much appreciate a correction if this is not the case. Finally, the Antibiotic dataset only includes concentrations after estimation from 16S and qPCR data, not the raw data itself.

We also now include gLV with parameters estimated using relative abundances in the model comparison (section 2.5, Figure 4). This is equivalent to assuming constant biomass (see below), eliminating a source of noise.

2. **Section 2.2 provides some good intuition on the connection between the proposed model and standard gLV.**

**[2.2.a]**

**However, it would be helpful if the authors provided some intuition on why the assumption of low variance of Var[N(t)] is essential. Can a model of dynamics over relative abundances still be defined or is essential information missing? Is the assumption more to make inference easier? Or is it to facilitate interpretation/comparison to standard gLV?**

[2.2.a] The assumption of low variance is only essential for the parameters of the two models to correspond. We note that mathematically equation (5) defines its own dynamical system, independent of gLV. The derivation provides motivation for the specific form of equation (5). We have revised section 2.2 to clarify these points (lines 155-166).

**[2.2.b]**

**Can anything be said (quantitatively) about what Var[N(t)] values will be low enough to provide a good approximation? The authors state the antibiotic dataset has a Var[N(t)] = 1.1, but it’s unclear what that means. Will that scale of variation result in an accurate approximation per equation (5)?**

[2.2.b] We now provide a heuristic for quantifying how low the variance should be for a good approximation (section 2.2, lines 155-161). In particular, we suggest a type of “signal-to-noise” ratio of the mean to standard deviation. This suggests that the approximation should be strong if Var[N(t)] < 1 after rescaling to E[N(t)]=1. In section 2.4 we provide empirical evidence in favor of our formulation.

**[2.2.c]**

**Also, with regards to alr and related transformations, can the authors address how feasible it is to find a taxon as a “reference” to use (e.g., x_D) in human datasets? In many human datasets, there may be no taxon that’s consistently present across all human subjects. Realistically, how much will the smoothing assumptions as described in Section 4.6 impact such datasets? Again, I understand that some of those issues have been addressed in prior compositional literature on the microbiome, but since alr is the main transformation discussed, it’s important to make some of these limitations clear upfront.**

[2.2.c] Our understanding of the current state of the compositional literature is that there are methods to deal with count zeros, but not essential zeros. That is, there are methods to deal with observations that fall below the detection threshold, but not absence from the community completely. Additionally, the reviewer is correct that finding a taxon as a reference may be challenging in human datasets. We have revised the discussion to highlight this limitation (lines 392-401).

3. **The section on parameter inference 2.3 seems fairly superficial and not particularly well justified. I understand the primary purpose of this section was to show that elastic-net regularization is better than other methods for this application, but I still think some more justification is needed. Also, including comparisons to OLS seems irrelevant, since prior work in the field has used ridge regression. Empirical studies presented in prior work (and theory) argue that many datasets analyzed lack sufficient information to infer parameters without regularization. So, I think comparisons should be elastic net to ridge regression, not to OLS. Regarding the synthetic data used for testing, the authors state in the Methods (line 369) that “We choose these parameters because simulated trajectories were qualitatively similar to observed trajectories on real data.” In what sense? Did they investigate a range of different parameters and similarly show robustness of their inference method? Also, the authors should provide some justification for their measurement noise model (lognormal Poisson) for simulating data; other groups have used other models including negative binomial and zero-inflated models. While I don’t think the authors need to evaluate every possible noise model, since this isn’t the central thrust of their work, they should at least justify why they used the particular model they did. Another issue is that elastic-net and ridge regression aren’t the only possibilities for inference, and several alternate methods have been demonstrated to be superior in prior literature. These include Bayesian variable selection methods (Bucci et al), which has been demonstrated to outperform regularization on many of the same datasets the authors are evaluating. Again, while I understand that the primary purpose of this study wasn’t to evaluate a set of inference methods, the authors should be clearer about the limited scope of their investigations into parameter inference for their model.**

[2.3] We redesigned our simulation framework to address these concerns. We now follow the simulation framework described in Bucci *et al.*, including their measurement noise model using the Dirichlet-Multinomial distribution. We explore parameter inference performance with respect to sample size, density in time, and sequencing depth across a larger number of simulations than before. In addition, we now compare elastic net regularization to ridge regression (section 2.3).

We note that directly applying some of the more sophisticated methods proposed by Bucci *et al* to our model is not immediately straightforward. This is because of the form of equation (6) has additional terms compared with gLV. We discuss this briefly in the manuscript.

4. **In analyses of the real datasets, there are several issues.**

**[2.4.a]**

**The authors state that their method performs best when variance of the total abundance is lower, which is theoretically justified based on their modeling assumptions. They later state in the Discussion that total abundance measurements may be quite noisy. Both these factors were recognized in Bucci et al’s work (Gen Biol 2016) and they performed an analysis testing whether an assumption of constant biomass would change inferences and predictions, indeed on two of the same datasets analyzed in the present manuscript (Diet and C. diff.) The conclusion of Bucci et al was that the assumption of constant biomass was least justifiable for the C. diff dataset. Given all these findings, and if the authors’ main question is prediction of relative abundances, would assuming constant biomass in the gLV model indeed lead to more accurate prediction of RELATIVE ABUNDANCES than using the biomass data? Since the authors’ assume measurements are noise-free in their model, this seems important to at least try.**

[2.4.a] We note that the performance of cLV is not *a priori* dependent on variance in community size, only the correspondence between its parameters and gLV.

To test whether assuming constant biomass would lead to more accurate predictions, we now included gLV with parameters estimated using relative abundances in our model comparison. This is equivalent to assuming constant biomass. We note that predictions by cLV were more accurate than predictions by gLV using relative abundances (lines 258-260, Figure 4).

**[2.4.b.i]**

**As I understand it, C. diff is being treated as an on-off perturbation. Why? It’s clearly a time-varying change in the ecosystem. The previous work from which the present authors obtained the data treated C. diff as another organism in the ecosystem that was introduced later in the time-course. This would seem to make most sense and could well explain why “Both models performed similarly on the C. diff dataset, but neither captured a community disturbance due to the introduction of C. diff.” (starting on line 232.).**

[2.4.b.i] We included *C diff* in the model in addition to treating its introduction as external perturbation. We reasoned that the act of introducing *C diff* may have an impact on the community beyond the effect of the taxon itself. We have clarified this in two places: line 232 and lines 466-467.

**[2.4.b.ii]**

**This is discussed somewhat in the Discussion section, but it seems a bit odd to analyze a data set using a method that can’t capture a central feature of the biological system under study (invasion of a pathogen.) In the Discussion section the authors state that “A further fundamental limitation of all models based on log-ratios is the inability to describe extinction and colonization. Each taxon is assumed to exist at each time point.” Can the authors elaborate on this? It’s not clear to me why this is true, since they use a smoothing method that effectively replaces zeros in the data. Is the inability to describe extinction and colonization an absolute limitation of log- ratio based models or just an issue of less accuracy?**

[2.4.b.ii] Methods in compositional data analysis have ways of dealing with count zeros, where a taxon falls below the detection threshold, but not essential zeros. Implicitly, this means assuming that all taxon exist at all time points. We expand on this point in the Discussion (lines 392-396).

**[2.4.b.iii]**

**Also, in the Methods section the authors describe excluding 6 taxa from their analyses. This wasn’t done in the original Bucci et al work. Why did the authors need to do so here? Did it have to do with their not having a noise-model and therefore not being able to handle lower abundance species? Could excluding all these organisms (~50% of the community) also account for their not being able to see a “community disturbance due to the introduction of C. diff?”’**

[2.4.b.iii] In the count matrices in the supplement of Bucci et al, there are 23 taxa total. Two taxa have zeros for >99% of their observations, three with >91% zeros, and one with 86% zeros. We originally felt that these were not informative enough to learn dynamics and had excluded them.

Nonetheless, in light of concerns about how noise could affect parameter estimates, we ran MDSINE on this dataset and extracted the denoised estimates on this dataset. As far as we can tell, MDSINE performs filtering similar to our original approach (line 62 in doBayesianSelect.m). Of the original 23 taxa only 14 are retained for inference.

**[2.4.c]**

**For the allo-HSCT patient data, the authors don’t compare performance of their method with the others on the Enteroccocus prediction task. Would the linear methods or gVL (with assumption of constant biomass) yield similar AUCs and/or interpretable interactions? Since the theme of the paper seems to be comparisons between these methods, comparing performance of the other methods on the same task seems relevant. Also, I’m not sure what’s meant by a strong positive effect of Lachnoclostridium on Enterococcus and Bacteroides on Enterococcus. Since the model can’t distinguish direct from indirect effects, as stated in the Discussion section, how are these effects to be interpreted?**

[2.4.c.] We have replaced this section with a new section 2.6 on interpreting model parameters. In particular, we provide a proof-of-concept for when direct effects can be recapitulated from compositional data.

5. **Minor issues or comments:**

**[2.5.a]**

**Starting on line 50: “However, gLV-based models describe dynamics in terms of absolute densities of taxa and require measurements of community size—either from quantitative PCR or spiked-in samples of known concentrations—in addition to sequencing counts of constituent taxa (Cao et al., 2017).” FACS or other cell-counting methods are another possible way to estimate the total community size, and should be mentioned.**

[2.5.a] We have made this change.

**[2.5.b]**

**Starting on line 67 “Sequencing counts only contain information about the relative abundances of community members: the total number of sequencing reads is independent of the size of the community, and relative abundances only provide information about how the proportions of each species change over time.” It’s true that the total number of sequencing reads is independent of the size of the community. However, it’s also important to stress that sequencing reads are what’s actually being measured, NOT relative abundances. Indeed, sequencing reads introduce an additional layer of variability that need to be accounted for to properly model the data.**

[2.5.b] This now reads: “Sequencing counts only contain noisy information about the relative abundances of community members:  the total number of sequencing reads is independent of the size of the community.”
